# Schizoaffective disorder induced by substance abuse: a case report

**DOI:** 10.1192/j.eurpsy.2022.2150

**Published:** 2022-09-01

**Authors:** A. García Carpintero, B. Rodado León, A. Bermejo Pastor, M. Jiménez Cabañas, T. Ponte López

**Affiliations:** 1 Hospital Clínico San Carlos, Instituto De Psiquiatría Y Salud Mental, Madrid, Spain; 2 Proyecto Hombre, Centro De Rehabilitación Y Tratamiento De Drogodependientes, Madrid, Spain

**Keywords:** Cannabis use, cocaine use, Schizoaffective disorder

## Abstract

**Introduction:**

We present the case of a 33-year old man that suffer chronic cocaine and cannabis use since adolescence and at age of 25 develops depressive symptoms and later psychotic symptoms not congruent with mood state. He met criteria for schizoaffective disorder at that moment and was treated with antidepressants and antipsychotic drugs, improving symptomatology even without stopping completely substance use.

**Objectives:**

To study the relationship between schizoaffective disorder and cannabis and cocaine use, including the neurobiological disturbance secondary to these drugs that can lead to the development of this disorder and the relevance of diagnosing it in context of active substance use.

**Methods:**

We carried out a literature review of scientific papers in Medline data base. We used the following terms: “Schizoaffective disorder” “cocaine use” and “cannabis use”. We considered English and Spanish papers for the last 5 years.

**Results:**

After 4 months of cocaine withdrawal and 1 month of cannabis withdrawal, the patient progressively improved depressive and positive psychotic symptoms. However, we reported the persistence of negative symptoms as psychomotor slowdown and cognitive and affective flattening.

**Conclusions:**

The use of cocaine and cannabis is related to depressive and psychotic symptoms in intoxication and can also precipitate chronic psychotic and affective disorders. Induced schizoaffective disorder has not been widely described in literature. Our patient could be a case of schizoaffective induced disorder, but we should consider other pathogenic factors, differential diagnosis and clinical evolution in permanent withdrawal to confirm this diagnosis.

**Disclosure:**

No significant relationships.

